# Development of ex vivo organ culture models to mimic human corneal scarring

**Published:** 2012-12-01

**Authors:** Hélène Janin-Manificat, Marie-Rose Rovère, Stéphane D. Galiacy, François Malecaze, David J.S. Hulmes, Catherine Moali, Odile Damour

**Affiliations:** 1 Banque de Tissus et Cellules, Hospices Civils de Lyon, Lyon, France; 2 Hôpital E. Herriot, Service d’Ophtalmologie, Lyon, France; 3 INSERM U563, Centre de Physiopathologie de Toulouse Purpan, CHU Purpan, Toulouse, France; 4 EA4555, Université Toulouse III Paul Sabatier, Toulouse, France; 5 CHU Toulouse, Hôpital Purpan, Service d’Ophtalmologie, Toulouse, France; 6 Institut de Biologie et Chimie des Protéines, CNRS/Université de Lyon FRE3310, Lyon, France

## Abstract

**Purpose:**

To develop ex vivo organ culture models of human corneal scarring suitable for pharmacological testing and the study of the molecular mechanisms leading to corneal haze after laser surgery or wounding.

**Methods:**

Corneas from human donors were cultured ex vivo for 30 days, either at the air-liquid interface (AL) or immersed (IM) in the culture medium. Histological features and immunofluorescence for fibronectin, tenascin C, thrombospondin-1, and α-smooth muscle actin were graded from 0 to 3 for control corneas and for corneas wounded with an excimer laser. The effects of adding 10 ng/ml transforming growth factor-β1 (TGF-β1) to the culture medium and of prior complete removal of the epithelium and limbus, thus preventing reepithelialization, were also analyzed on wounded corneas. Collagen III expression was detected with real-time PCR.

**Results:**

Wounding alone was sufficient to induce keratocyte activation and stromal disorganization, but it was only in the presence of added TGF-β1 that intense staining for fibronectin and tenascin C was found in the AL and IM models (as well as thrombospondin-1 in the AL model) and that α-smooth muscle actin became detectable. The scar-like appearance of the corneas was exacerbated when TGF-β1 was added and reepithelialization was prevented, resulting in the majority of corneas becoming opaque and marked upregulation of collagen III.

**Conclusions:**

The main features of corneal scarring were reproduced in these two complementary models: the AL model preserved differentiation of the epithelium and permits the topical application of active molecules, while the IM model ensures better perfusion by soluble compounds.

## Introduction

Corneal scarring is a commonly occurring consequence of several forms of trauma, e.g., wounds, chemical burns, infections, and refractive surgery. Since refractive surgery has become one of the most commonly used surgical procedures worldwide, it is now an important concern that in a small percentage of cases, wound repair results in the formation of a scar, commonly called “haze,” at the center of the cornea, which induces a loss of visual acuity. Therapeutic tools for preventing or treating corneal haze are presently limited [1-3], thus raising an urgent need to better understand the mechanisms involved in postoperative recovery.

Epithelial lesions normally resolve within several days, without any fibrotic response, due to the migration of stem cells from the surrounding limbus [4,5] or from other parts of the epithelium [6,7] into the injured region. Only in the case of disruption of the epithelial basement membrane can pathological evolution of the healing process occur, due mainly to fibrogenic factors, such as transforming growth factor-β1 (TGF-β1) and TGF-β2 [8], released by the injured epithelial cells [9,10], and inflammatory cells [11], into the stroma. TGF-β2 is also present in tear fluid [12]. These growth factors (and others) trigger the activation of quiescent stromal cells (keratocytes) from the wound periphery that then repopulate the wound area where resident cells have died by apoptosis soon after injury [13]. These activated keratocytes proliferate and undergo phenotypic changes typical of myofibroblasts [14] with increased ability to synthesize the extracellular matrix and promote wound contraction, as evidenced by the expression of α-smooth muscle actin (α-SMA). In addition, myofibroblasts themselves produce TGF-β [15], thus amplifying the response.

A related consequence of injury is that the turnover of the extracellular matrix is accelerated [16]. Among the proteins abundantly expressed during the first steps of wound healing are cellular fibronectin [17,18] and collagen III [19]. At later stages, the provisional matrix is replaced by a tissue rich in collagen I with functional properties (fibril diameter, orientation, and lamellar organization) similar to those of the initial tissue. In the case of pathological evolution, markers of the provisional matrix persist, and myofibroblasts do not die by apoptosis as expected. Other matrix proteins are known to play important roles during corneal wound healing, especially the proteins that modulate cell-matrix interactions such as tenascin C and thrombospondin-1. Tenascin C is transiently expressed in the wound periphery [18] and is thought to control fibroblast recruitment to the wound area [20]. Its persistence is a hallmark of a fibrotic process. Thrombospondin-1 is known to accelerate corneal reepithelialization after epithelial injury [21] and to inhibit neoangiogenesis [22], thus contributing to maintaining corneal transparency.

Several in vivo and ex vivo models of corneal wound healing have been developed in rodents and rabbits with various types of wounds: mechanical [10,23,24], alkali burns [25], and excimer laser [26,27]. However, rodents heal faster than humans with a reduced tendency to develop scars. In this respect, rabbits are more similar to humans, but the panel of available tools (gene sequences, antibodies, small interfering RNAs, etc.) is reduced. In addition, the differences in Bowman’s membrane and stromal organization between rodents, rats, and humans [28] could potentially lead to differences in repair mechanisms. Thus, there is a need to develop ex vivo models of human corneal scarring, capable of reproducing as many features as possible of the in vivo situation, for testing novel therapeutic agents. Several such ex vivo models have been described [29-35], though these are mainly concerned with the process of reepithelialization, and only limited studies on markers of wound healing have been performed [30,34,36]. In particular, none of the ex vivo models described has been shown to develop the opacity that is characteristic of corneal scarring or haze.

Here we describe two ex vivo models of corneal scarring, using human donor corneas rejected for keratoplasty. Wounds were created using an excimer laser, to mimic refractive corneal surgery, and then corneas were cultured in either immersed conditions or raised to the air-liquid interface, for 30 days. We tested the effects of the epithelium/limbus and of exogenous TGF-β1 on the wound healing process, with histology and immunofluorescence for markers of keratocyte differentiation (α-smooth muscle actin) and wound healing (fibronectin, tenascin C, thrombospondin-1), and with real-time PCR analysis for expression of collagen III. For the first time, we established conditions leading to the development of corneal opacity (absence of epithelium/limbus and presence of exogenous TGF-β1). In addition, we obtained models of intermediate severity when one or more of these parameters were changed. This system therefore appears sufficiently versatile for testing novel therapeutic agents.

## Methods

### Cornea collection from the cornea bank

Corneas from human donors (ages 40–80) were collected in agreement with the tenets of the Declaration of Helsinki, including the consent of families for clinical and scientific use, and then stored in the cornea bank of the Hôpital E. Herriot (Lyon, France), at 31 °C in organ culture medium, for a maximum of 21 days before use. Since corneas were collected for grafting purposes, patient selection followed a rigorous set of criteria, as defined by the French Agence de la Biomedicine in conjunction with the Hospices Civils de Lyon. These include absence of viral, bacterial, or parasite infections, low risk of Creutzfeldt-Jacob disease, absence of tumors, lack of treatment with immunosuppressants or blood products, unknown cause of death, lack of exposure to toxic substances, and absence of corneal pathology or corneal surgery. The corneas used for the experiments described here were rejected for grafting because of low endothelial density.

### Epithelium removal and excimer laser treatment

**Figure 1:**
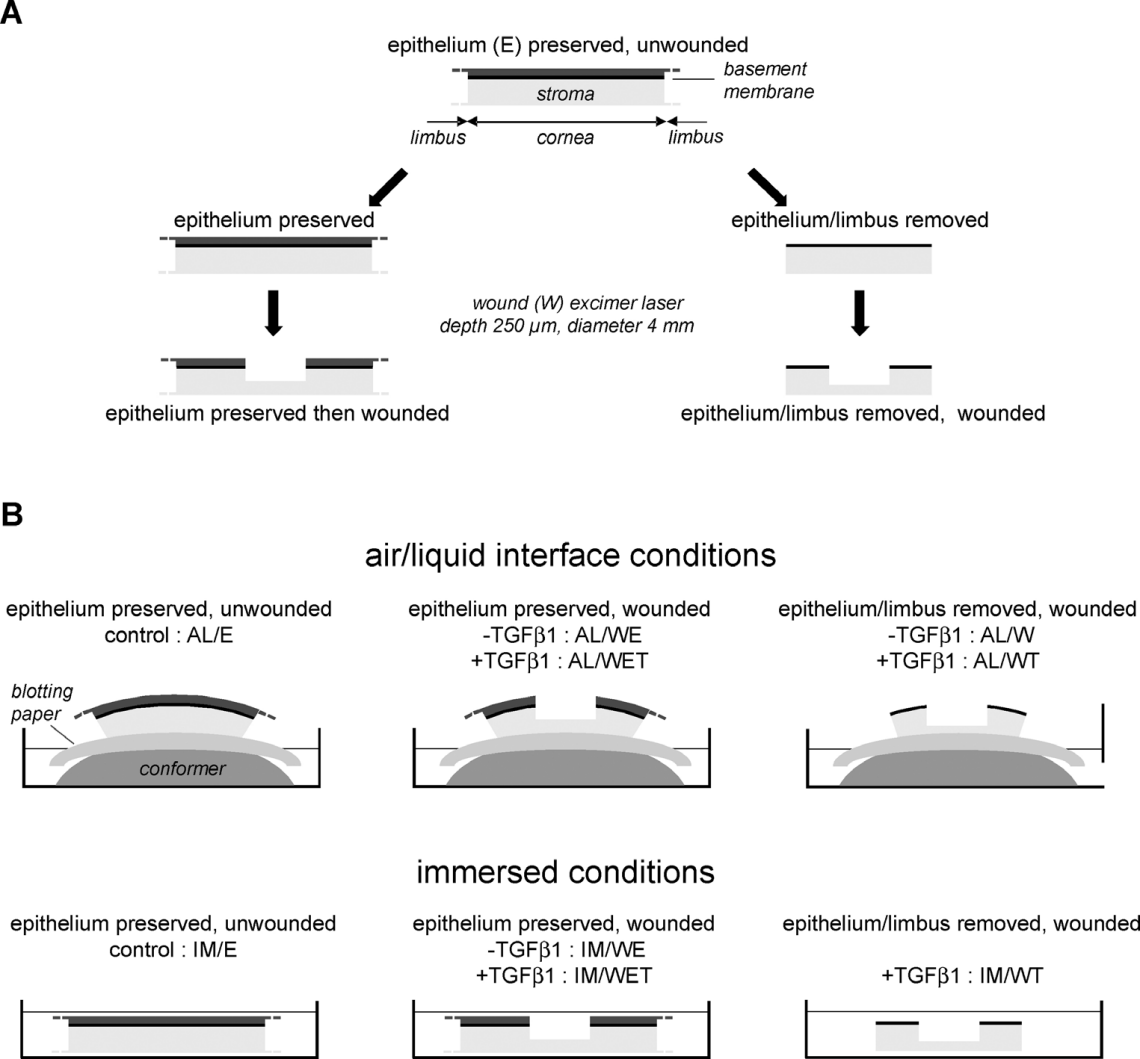
Conditions tested in this study. **A**: Details of procedures performed before subsequent culture, including removal of the epithelium and the outer part of the cornea including the limbus, leaving the epithelial basement membrane intact, and wounding using an excimer laser. **B**: Diagrammatic representation of the different culture conditions used, at the air-liquid interface (AL) and fully immersed in culture medium (IM). Conditions include non-wounded controls with the epithelium and limbus intact (represented by E) and wounded corneas (W) with and without prior removal of the epithelium and limbus, and with and without TGF-β1 (T) added at a concentration of 10 ng/ml.

Depending on the conditions studied, the corneal epithelium and limbus (denoted collectively by E in Figure 1) were sometimes removed before culture. In these cases (the W and WT conditions), the epithelium was peeled off using a 6 mm lancet shaped Desmarres-Paufique keratoplasty dissector knife (Moria, Antony, France) in such a way as to leave the basement membrane intact, and the limbus was removed using scissors or a trepan (8 mm). Wounding (denoted by W in Figure 1) was performed using an excimer laser (diameter 4 mm, depth 250 µm).

### Ex vivo cultures

Corneas were cultured, for 30 days at 37 °C in 5% CO_2_ using 10 ml of medium per six-well plate, either fully immersed (IM; epithelium uppermost; Figure 1) or maintained at the air-liquid interface (AL; Figure 1). Medium composition was as follows: Dulbecco's Modified Eagle Medium (DMEM)/Glutamax and Ham’s F-12 (both from GIBCO/Life Technologies, Saint Aubin, France) mixed in a 1:1 ratio with 10% fetal bovine serum (Hyclone/Thermo Fisher Scientific, Brebières, France), 50 µg/ml vitamin C (Bayer, Berlin, Germany), 100 UI/ml penicillin, 20 µg/ml streptomycin (Panpharma, Fougères, France), and 1 µg/ml Fungizone (Bristol Myers Squibb, Rueil-Malmaison, France). The medium was changed three times a week. In some conditions (denoted by T in Figure 1), the medium was supplemented with 10 ng/ml TGF-β1 (R & D Systems Europe, Lille, France). When cultured at the air-liquid interface, the posterior (“endothelial”) face of the cornea was in contact with sterile blotting paper, soaked with culture medium, and placed on a conformer to maintain the curved shape of the cornea, while the anterior (“epithelial”) face was in contact with air (Figure 1). A drop of medium was added twice a day either to the top of the cornea or directly into the wound (when present) to mimic the effect of tears.

### Histological analyses and immunofluorescence

After 30 days in culture, corneas were cut into two halves: the first was fixed with formalin before paraffin inclusion while the second was frozen with optimal cutting temperature embedding medium (Tissue Tek). Histology was performed after hematoxylin phloxine saffron (HPS) staining. For immunofluorescence, frozen sections (5 µm) were first blocked in phosphate buffered saline containing 1% (w/v) bovine serum albumin and 5% normal goat serum. Mouse monoclonal primary antibodies were used as follows: anti-α-SMA (Novocastra, Newcastle-upon-Tyne, UK; dilution 1:100), antitenascin C (Dako, Trappes, France; dilution 1:200), antifibronectin (Santa Cruz Biotechnology, Heidelberg, Germany; dilution 1:50), antithrombospondin 1 (Thermo Fisher; dilution 1:100). The secondary antibody was either fluorescein isothiocyanate–conjugated sheep antimouse immunoglobulin G (IgG) (TEBU Bio, Le Perray en Yvelines, France; dilution 1:100) or Alexa Fluor 488-conjugated goat antimouse IgG (Invitrogen/Life Technologies, Saint Aubin, France; dilution 1:100). Control IgG was from Abcam (Paris, France). Nuclei were stained with 4',6-diamidino-2-phenylindole (DAPI) using Vectashield mounting media for fluorescence (from Eurobio/Abcys, Les Ulis, France). Photos were taken with an Eclipse 50i optical microscope using NIS-Elements software (Nikon, Champigny sur Marne, France).

Immunostaining for collagen III was attempted using the following antibodies: goat anticollagen type III (Southern Biotech, Birmingham, AL) and mouse anticollagen type III (Novotec, Lyon, France). However, although positive results on control human skin samples were obtained, the signal observed with human corneas was much less satisfactory. Thus, collagen III was assessed indirectly by measuring RNA levels with real-time PCR (see below).

Semiquantitative evaluation of immunofluorescence was performed by three observers independently using a scale from 0 to 3, where 0=no staining in the wound area (or central area when no wound was present), 1=presence of stained dots in the wound area, 2=marked staining of localized regions of the wound, and 3=intense/uniform staining all over the wound area. Similarly, the scale used for histology was 0=no stromal disorganization, 1=weak stromal disorganization/few activated keratocytes, 2=moderate stromal disorganization/activated keratocytes, and 3=strong stromal disorganization/numerous activated keratocytes. Statistical significance was determined using the Mann–Whitney test, using a p value of <0.05.

### Real-time polymerase chain reaction detection of collagen III

Total RNA was extracted from corneal stromas (after the epithelium was removed when present) following culture in the IM/E condition (controls; six samples) and the IM/WT condition (wounding and TGF-β1 in the absence of the epithelium and limbus; six samples). Corneas were then frozen and transferred from liquid nitrogen to 500 µl RLT buffer (RNeasy Mini kit, Qiagen, Courtaboeuf, France)/1% β-mercaptoethanol mix and kept on ice. This mix was then subjected to eight cycles of 20 s shaking followed by 5 min of cooling at 4 °C on a FastPrep-24 System (MP Biomedicals, Illkirch, France). The supernatant was then retrieved, and total RNA was extracted and further purified using an RNeasy Mini kit and RNase-Free DNase set (Qiagen, Courtaboeuf, France). RNA integrity was assessed with a 2000 Nano Kit (Agilent, Massy, France); each sample used had an RNA integrity number of at least 7. Reverse transcriptase PCR was performed using the Invitrogen Superscript III VILO kit according to the manufacturer’s recommendations. Real-time PCR was performed on 2.5 ng cDNA in a Roche (Meylan, France) LightCycler 480 using Roche SYBR Green I Master mix. Primers were from Eurogentec (Angers, France; COL3A1 sense: 5’-GGT GCT CGG GGT AAT GAC G-3’; COL3A1 antisense: 5’-TCC AGG GAA TCC GGC AGT T-3’). Three genes, already validated for human corneas [37], were used as standard baseline genes: TATA box binding protein, fibrosin, and PIH1 domain-containing protein 1. PCR efficiency was determined for each primer set to calculate the expression ratio. The fold-change in gene expression was calculated using the 2-ΔΔCT ratio. PCR products were checked with sequencing (Millegen, Labege, France).

### Evaluation of corneal transparency

Corneal transparency was evaluated, after 15 and 30 days, by reading text written in Arial characters (8-point font size) through the cornea. Corneas were classified as “opaque” when the text could not be seen through the cornea.

## Results

Using two different experimental configurations, one closer to the in vivo situation (corneas cultured at the air-liquid interface, denoted by AL in Figure 1) and one that favored diffusion of components from the culture medium (immersed corneas, denoted by IM in Figure 1), we analyzed the effect of three parameters (wounding, addition of TGF-β1, presence of epithelium/limbus) for their ability to promote a corneal scar-like response. This was evaluated in terms of stromal organization and number of activated keratocytes (HPS staining), neosynthesis of extracellular matrix components (immunofluorescence for fibronectin), expression of specific markers of wound healing (immunofluorescence for tenascin C and thrombospondin-1), presence of myofibroblasts (immunofluorescence for α-SMA), and transparency (visibility of Arial 8-point characters). Since collagen III expression could not be detected with immunofluorescence (see Methods), this was evaluated indirectly with real-time PCR.

### Air-liquid interface model

**Figure 2:**
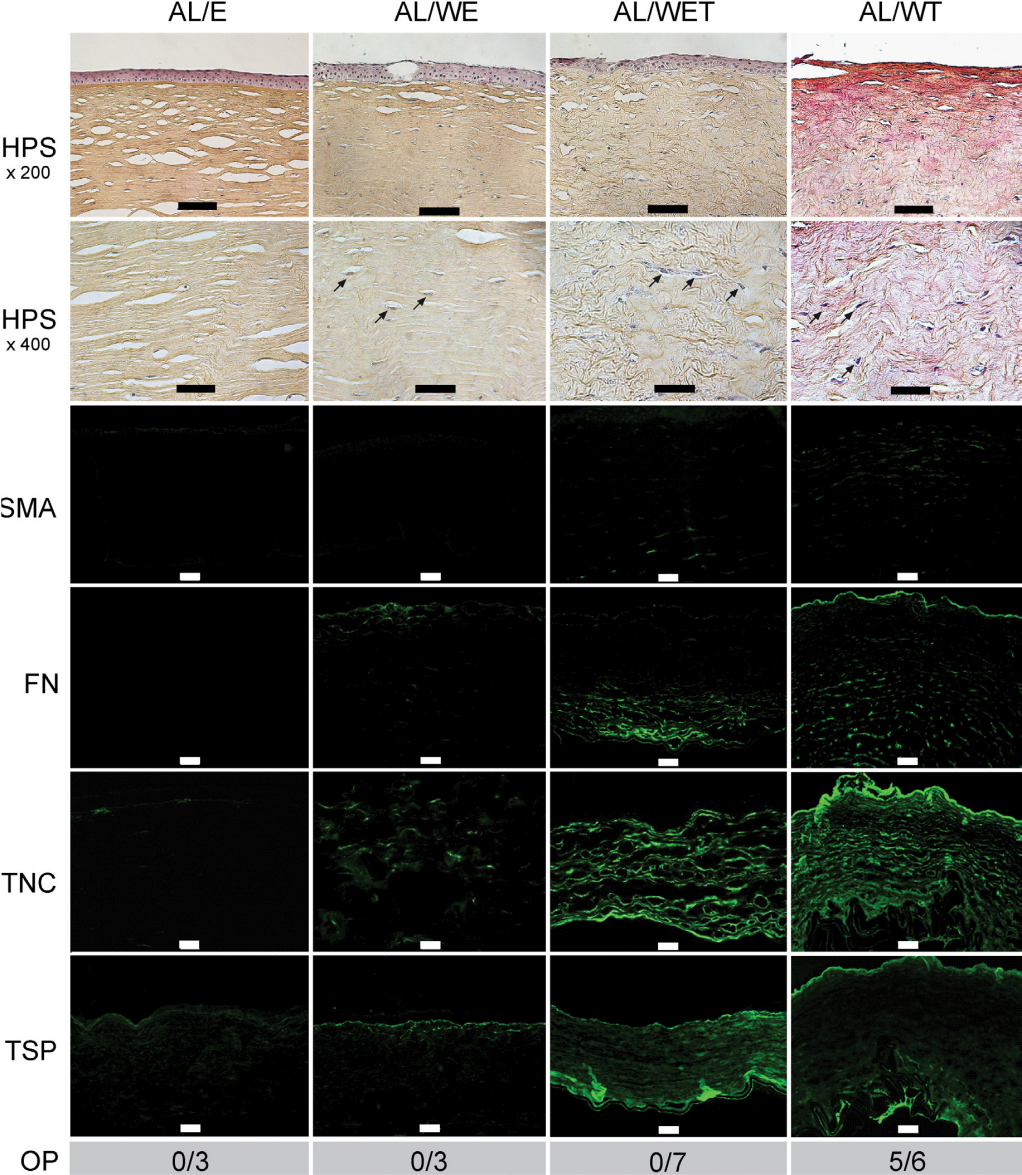
Corneas cultured at the air-liquid interface. For each condition (E=control with the epithelium and the limbus intact; WE=wounded without prior removal of the epithelium and limbus; WET=as WE but with TGF-β1 added at 10 ng/ml; WT=wounded after prior removal of the epithelium and limbus plus TGF-β1 added at 10 ng/ml), keratocyte activation (see arrows) and stromal disorganization were assessed with hematoxylin phloxine saffron (HPS) staining (scale bars: 100 µm (row 1), 50 µm (row 2)), expression of α-smooth muscle actin (SMA), fibronectin (FN), tenascin C (TNC) and thrombospondin-1 (TSP) by immunofluorescence (scale bars=100 µm), and corneal opacity (OP) by the ability to read Arial 8 characters (number of opaque corneas/total number of corneas examined for each condition). Each figure shows the wound region only, except the control E, which shows the central part of the cornea.

Figure 2 (the AL/E column) shows the characterization of human control corneas cultured for 1 month at the air-liquid interface. With HPS staining, the stroma showed the lamellar organization typical of the cornea, and the epithelium remained differentiated and pluristratified. No immunofluorescence for α-SMA or fibronectin was detected. Immunofluorescence for tenascin C was sparse. Immunofluorescence for thrombospondin-1 occurred in a punctate manner throughout the cornea with a slightly more intense signal beneath the basement membrane. None of these control corneas were opaque.

**Figure 3:**
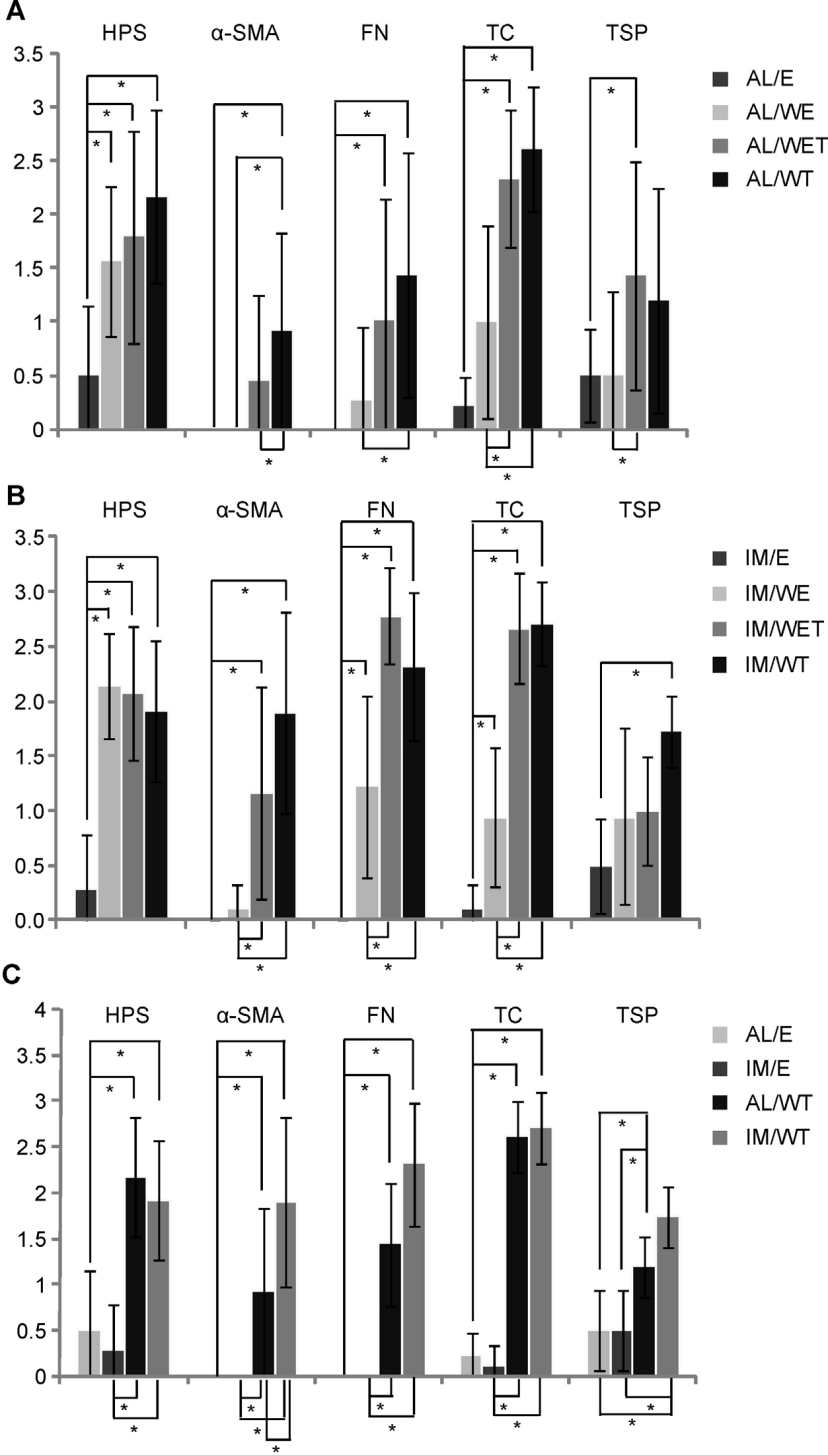
Semiquantitative evaluation of histochemical and immunofluorescence data. **A**: Corneas cultured at the air-liquid interface. The total numbers of corneas examined per group were: E=3, WE=3, WET=7; WT=6. **B**: Corneas cultured in immersed conditions. The total numbers of corneas examined per group were: E=3, WE=3, WET=3; WT=4. **C**: Comparison of semiquantitative data for control E and wounded corneas (WT; wounded after prior removal of the epithelium and limbus then cultured in the presence of added TGF-β1) between the air-liquid and immersed conditions. The total numbers of corneas examined per group were: AL/E=3, IM/E=3, AL/WT=6; IM/WT=4. In all cases, significant differences are indicated by * (p<0.05).

To determine if the wound by itself was sufficient to trigger a change in the histological appearance of the cornea or in the expression of the usual markers of wound healing, we compared the control corneas (AL/E) with the wounded corneas (AL/WE) cultured in the absence of added TGF-β1. Fluorescein staining indicated that complete reepithelialization was obtained after 7 days in wounded corneas, resulting in a pluristratified (five to six layers) epithelium. Bowman’s membrane was not reconstituted, as found in in vivo wounds [38,39]. Using HPS staining, we observed an increase in the number of activated keratocytes, as well as some stromal disorganization (Figure 2, the AL/WE column). Semiquantitative evaluation of several samples (Figure 3A) showed this increase was significant. Immunofluorescence for fibronectin and tenascin C also began to appear (Figure 2, the AL/WE column), but these changes were not significant (Figure 3A). In contrast, expression of thrombospondin-1 was unchanged (compared to the AL/E control) and α-SMA remained undetectable (Figure 2). Wounding alone did not result in corneal opacity.

Since the response to injury alone did not recapitulate all the features of wound healing and scarring, especially the lack of opacity and the failure of the keratocytes to differentiate into α-SMA-expressing myofibroblasts, we next determined the effects of adding TGF-β1 to the ex vivo cultured corneas. TGF-β1 was added to the medium at a concentration of 10 ng/ml. Using a single non-wounded control cornea, addition of TGF-β1 promoted limited expression of α-SMA, only in regions in direct contact with the culture medium (data not shown). In contrast, when the wound and TGF-β1 were present, α-SMA began to be expressed in the stroma (Figure 2, the AL/WET column), but this increase was not significant (Figure 3A). In addition, keratocyte activation was again observed (as for the AL/WE condition) as well as marked stromal disorganization, while the number of cell layers in the epithelium was slightly decreased in the presence of TGF-β1, in agreement with previous reports [24]. Adding TGF-β1 also led to marked increases in the expression of fibronectin, tenascin C, and thrombospondin-1 (compared to the AL/WE condition, Figure 2). In contrast, none of the seven corneas studied in this condition showed any sign of opacity, as found in the absence of TGF-β1 (the AL/E and AL/WE conditions).

**Figure 4:**
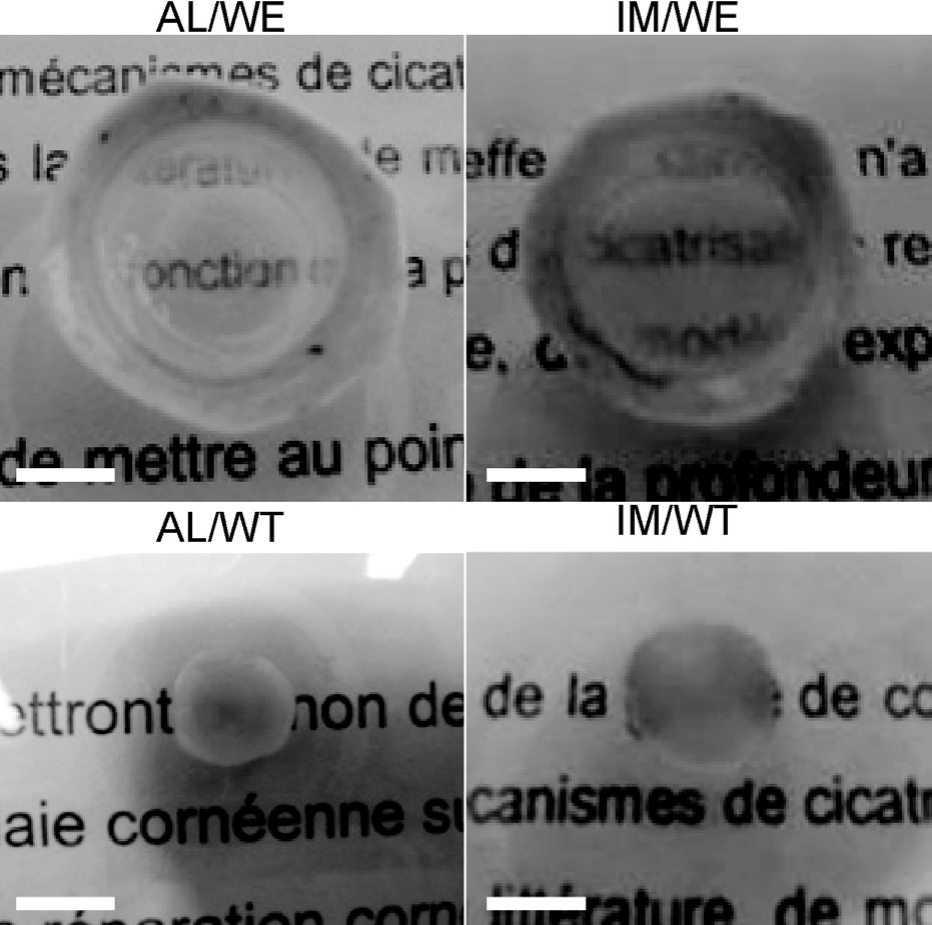
Assessment of corneal opacity. While corneas wounded without prior removal of the epithelium and the limbus (AL/WE, IM/WE) are transparent, corneas wounded after prior removal of the epithelium and limbus and cultured with added TGF-β1 (AL/WT, IM/WT) are opaque. Note that the latter corneas are smaller in diameter due to the total elimination of the limbus by trepanation (8 mm diameter trepan). Scale bars=5 mm.

We then tested the effect of removing the epithelium and limbus before wounding on the repair process (the AL/WT condition; Figure 1). Interestingly, in this condition, following wounding and the addition of TGF-β1, α-SMA expression was now significantly upregulated (Figure 2, the AL/WT column; Figure 3A) compared to the control condition (AL/E) and the AL/WE and AL/WET conditions. As also found with the AL/WET condition, all other parameters (keratocyte activation and stromal disorganization, expression of fibronectin, tenascin C, and thrombospondin-1) remained highly elevated (Figure 2; Figure 3A). Most strikingly, for the first time, five of the six corneas used for this condition were opaque, as illustrated in Figure 4. In contrast, in the absence of added TGF-β1, these corneas remained transparent (the AL/W condition; data not shown). In addition, even in the presence of added TGF-β1, no opacity was seen after 15 days in culture (data not shown), indicating that haze first appeared between 15 and 30 days.

### Immersed model

**Figure 5:**
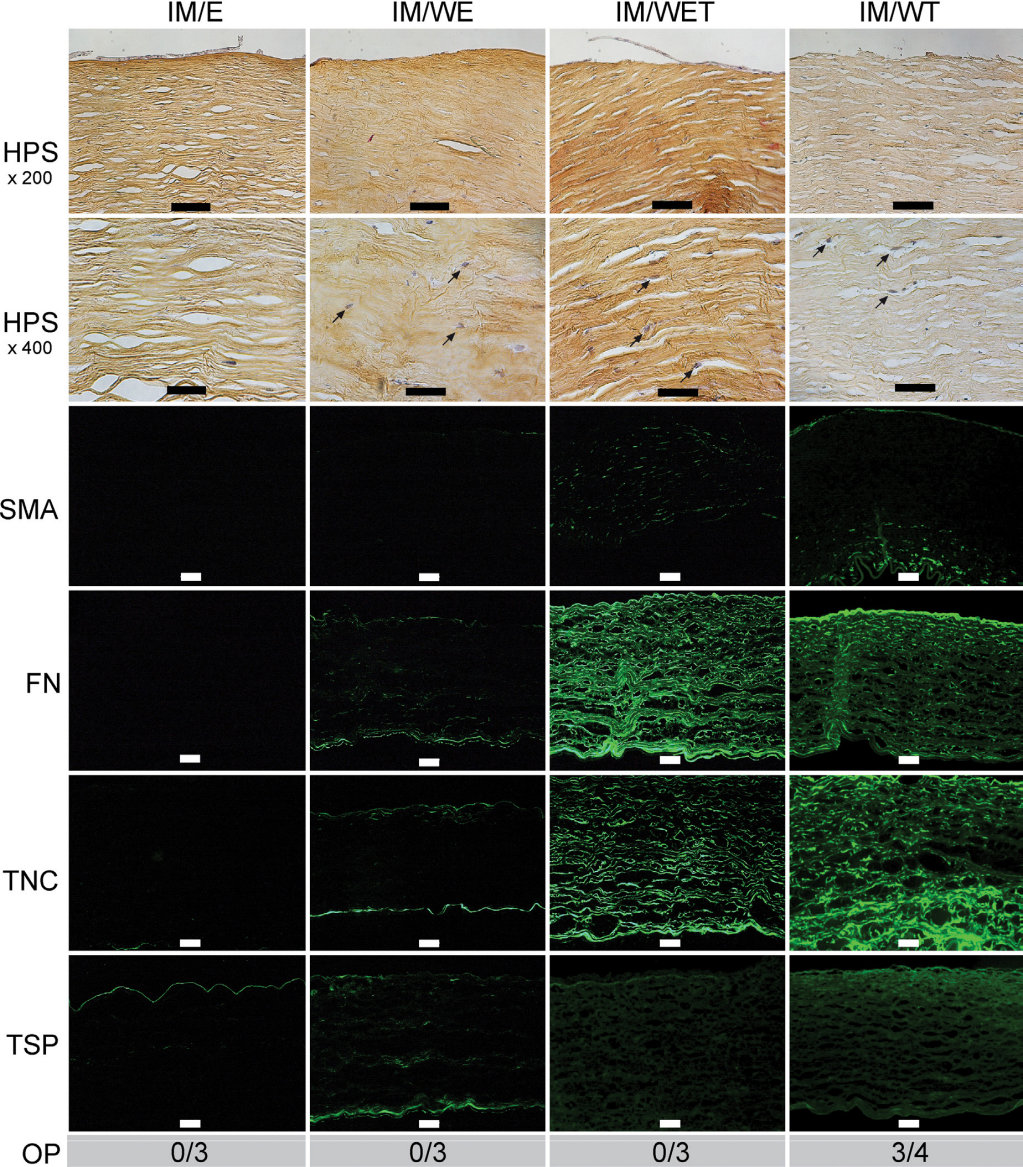
Corneas cultured in fully immersed conditions. For each condition (E=control with the epithelium and limbus intact; WE=wounded without prior removal of the epithelium and limbus; WET=as WE but with TGF-β1 added at 10 ng/ml; WT=wounded after prior removal of the epithelium and limbus plus TGF-β1 added at 10 ng/ml), keratocyte activation (see arrows) and stromal disorganization were assessed with HPS staining (scale bars: 100 µm (row 1), 50 µm (row 2)), expression of α-smooth muscle actin (SMA), fibronectin (FN), tenascin C (TNC), and thrombospondin-1 (TSP) with immunofluorescence (scale bars=100 µm), and corneal opacity (OP) by the ability to read Arial 8 characters (number of opaque corneas / total number of corneas examined for each condition). Each figure shows the wound region only, except the control E, which shows the central part of the cornea.

When corneas were cultured in IM conditions, in all cases the epithelium was reduced in thickness, as previously reported [33,40], and generally consisted of only one cell layer (Figure 5). In control conditions (Figure 5, column IM/E), the stroma showed a typical lamellar organization interspersed with mostly quiescent keratocytes. Furthermore, as with the air-liquid equivalent (Figure 2), no immunofluorescence staining for α-SMA, fibronectin, or tenascin C was detected. Weak staining was seen for thrombospondin-1, in the region of the epithelial basement membrane, as with the air-liquid equivalent. None of the three controls studied were opaque.

Following wounding, but permitting subsequent reepithelialization (the IM/WE condition), with HPS staining activated keratocytes and stromal disorganization/turgescence were seen (Figure 5; the IM/WE column). Semiquantitative analysis (Figure 3B) showed this was statistically significant. In addition, reepithelialization was complete after 7 days, as for the air-liquid equivalent, even though it consisted of a single cell layer, unlike the air-liquid equivalent. As with the air-liquid equivalent, α-SMA remained undetectable. Immunofluorescence staining for fibronectin and tenascin C was however greater than in the air-liquid equivalent, reaching statistical significance, compared to the controls (Figure 3B). Small increases in thrombospondin-1 staining were also observed (Figure 5), but these were not significant (Figure 3B).

Following wounding and with added TGF-β1 (the IM/WET condition), HPS staining continued to show keratocyte activation and stromal disorganization (Figure 5; the IM/WET column), though with less stromal disorganization than in the A/L conditions. As with the air-liquid equivalent, α-SMA began to be expressed in this immersed model, but in this case the increase was statistically significant (Figure 3B), compared to controls. Expression of thrombospondin-1 also increased (Figure 5) but not to a significant extent (Figure 3B). As with the air-liquid equivalent, none of the corneas were opaque.

Finally, as with the air-liquid model, when the epithelium and limbus were removed before wound healing, and in the presence of added TGF-β1, α-SMA expression in the immersed IM/WT condition was significantly upregulated (Figure 3B, Figure 5) compared to controls, as found in the air-liquid culture. Keratocyte activation and stromal disorganization were also seen (though with less stromal disorganization than in the A/L conditions), and fibronectin and tenascin C were strongly expressed, comparable to the IM/WET condition. Thrombospondin-1 was also highly expressed, reaching statistical significance. Most strikingly, however, as with the air-liquid equivalent, most of the corneas were now opaque, as illustrated in Figure 4.

We also investigated changes in the expression of collagen III. Since we were unable to obtain a satisfactorily specific signal with immunofluorescence, despite using different antibodies, we measured collagen III expression with real-time PCR. Collagen III mRNA was 175.7±61.8-fold more abundant in the stroma of corneas cultured in the IM/WT condition than in corneas cultured in the IM/E control condition.

### Comparison of the air-liquid interface and immersed models

Using air-liquid and immersed culture, the models for which wound healing occurred in the presence of added TGF-β1 and in the absence of the epithelium/limbus led to the most severe scars, characterized by corneal opacity, keratocyte activation, and stromal disorganization, with marked expression of α-SMA, fibronectin, tenascin C, and collagen III (immersed model). When the results of the semiquantitative analyses were compared, between control conditions (E) and those without epithelium plus TGF-β1 (WT), the same trends were apparent in the air-liquid and immersed cultures (Figure 3C). There were no significant differences between AL/E and IM/E, or between AL/WT and IM/WT, except in the case of α-SMA for which more intense staining was observed in the IM/WT condition compared to AL/WT. In conclusion, air-liquid and immersed cultures appeared suitable for reproducing most of the events of corneal scar formation after injury. A summary of the main findings of this study is shown in Table 1.

**Table 1:** Summary of the main findings presented in this study.

Air/liquid conditions	Epithelium/limbus preserved, unwounded	Epithelium/limbus preserved, wounded	Epithelium/limbus preserved, wounded, TGF-β1	Epithelium/limbus removed, wounded, TGF- β1
Keratocyte activation, stromal disorganization	-	+	++	++
Myofibroblast differentiation (α-SMA)	-	-	+	+
ECM production (fibronectin, tenascin C, thrombospondin-1)	-	+	++	++
Corneal opacity	-	-	-	+

Immersed conditions	Epithelium/limbus preserved, unwounded	Epithelium/limbus preserved, wounded	Epithelium/limbus preserved, wounded, TGF- β1	Epithelium/limbus removed, wounded, TGF- β1
Keratocyte activation, stromal disorganization	-	-	+	+
Myofibroblast differentiation (α-SMA)	-	-	+	+
ECM production (fibronectin, tenascin C, thrombospondin-1)	-	+	++	++
Corneal opacity	-	-	-	+

## Discussion

Using human corneas cultured in ex vivo conditions, we preserved the histological quality of the control corneas, for both the stroma and the epithelium, in the air-liquid configuration and for the stroma only in the immersed configuration, as well as their transparency. Importantly, we reproduced the loss of transparency observed in patients when corneas were wounded, after prior removal of the epithelium and limbus, and then cultured in the presence of added TGF-β1. Analysis of the expression of several extracellular proteins, combined with histological characterization, demonstrated the ability of the models to reproduce corneal scarring in terms of stromal disorganization, keratocyte proliferation, differentiation into myofibroblasts, and enhanced synthesis of provisional extracellular matrix components, especially fibronectin, tenascin C, and collagen III (albeit that the latter was detected only at the RNA level). As we used intact human corneas for these studies, the models described here are closer to the in vivo situation than the three-dimensional cell culture models reported in the literature [41,42]. Our models also extend previous ex vivo models [29-35] through the use of additional wound healing markers. Most importantly, to our knowledge, this is also the first time that a loss of transparency has been reported in an in vitro model. Although further optimization is possible, for example, by adding other inflammatory cytokines or fine-tuning the time scale of the ex vivo culture, these models already represent valuable tools for studying wound healing and for testing various compounds that might affect, positively or negatively, the healing process.

As expected, we found that wounding alone was not sufficient to trigger the scarring process in human ex vivo corneas, even though keratocyte activation and limited synthesis of fibronectin and tenascin C were already observed. Since this limited response was linked to the absence of inflammatory cells (normally infiltrating the wound from the limbus and from tear fluid), we added TGF-β1, the most well-known and widely used fibrogenic growth factor. As expected, this resulted in the appearance of α-SMA expression (albeit weak), in air-liquid and immersed conditions. Only when regeneration of the epithelium after wounding was prevented by prior removal of the epithelium and limbus was it possible to obtain opaque scar-like tissue. This is consistent with the known role of the epithelium in preventing scar formation [9]. These observations also suggest that regenerating the epithelium creates a barrier preventing diffusion of exogenous TGF-β1.

During the course of this study, different concentrations of TGF-β1 were tested. At low concentrations (1–2 ng/ml), no corneal opacity was observed (data not shown). Only at a concentration of 10 ng/ml did the corneas become opaque (in the absence of the epithelium). The choice of optimum TGF-β concentration has been discussed in the context of corneal keratocyte culture models [41,43], where 0.25 to 1 ng/ml was optimal. Higher doses (10 ng/ml) led to contraction of keratocyte culture sheets [41,44], as found when cells were cultured in collagen gels [45]. These reports raise the question of whether the opacity observed here, when the corneas were cultured in the presence of 10 ng/ml TGF-β and in the absence of the epithelium and limbus, could be due at least partly to retraction. Retraction might have occurred to some extent, as sometimes the final diameter of the corneas was less than that of the trepan (8 mm) used to isolate the central region (Figure 4). Though this parameter was not measured systematically, such effects were probably relatively small. The fact that this was not observed when intact corneas were used may be due to the extra rigidity provided by the surrounding scleral tissue.

Since the main objective of this study was generating stable corneal haze, we did not analyze stromal parameters on shorter time scales, though it is likely that our models could also provide useful information concerning the early steps of the healing process. Notably, it would certainly be informative to analyze the corneas that retained their epithelium, just before complete reepithelialization (5–6 days after wounding in our models) when the switch between inflammatory and “granulation” phases occurs in vivo and when epithelial-stromal interactions are maximal. Thrombospondin-1 especially might be expressed at a higher level as has been described in vivo after corneal injury [21,46]. In addition, since TGF-β2 plays a major role in controlling epithelial-stromal interactions during corneal repair [10], comparing TGF-β1 and TGF-β2 for their ability to promote a scar-like evolution of the wounded corneas would be interesting. Adding other fibrogenic cytokines (PDGF, CTGF, etc.) could also influence the scarring process and could be included in these models.

Although the link between haze and an abnormal repair process is clearly established, the mechanisms responsible for the loss of transparency are less well understood. In the models described here, two main parameters may have contributed to the loss of transparency: (i) the increased number of cells with altered morphology and altered refractive properties and (ii) the deposition of an excess of extracellular matrix molecules with inappropriate organization. Concerning the first parameter, changes in cell content and reduced expression of corneal crystallins observed in myofibroblasts compared to quiescent keratocytes has been shown to reduce corneal transparency [47,48]. More complex to analyze are the amount and architecture of the newly deposited extracellular matrix since, in the cornea, excessive matrix deposition has never been clearly linked to scarring as in skin, for example. Since there is no direct relationship between the apparent disorganization of collagen fibers and the persistence of corneal haze, however, increased keratocyte activation and disruption of the subepithelial layer seem to be the major contributory factors [49-51]. Further studies with transmission electron microscopy are required to address these questions.

Although the air-liquid interface and immersed models can reproduce corneal scarring, they also have specific advantages and disadvantages. The expression of some of the markers seemed higher in the immersed model; however, these increases proved statistically significant only for α-SMA. Nonetheless, perfusion of the cornea with soluble products (including growth factors and pharmacological agents) is expected to be more efficient in this model. In contrast, culture at the air-liquid interface maintains the differentiated epithelium and is closer to the in vivo situation. In addition, topical application of creams and other insoluble formulations could be tested in this model.

In conclusion, we have developed two ex vivo models of human corneal wound healing that (albeit different from the in vivo situation) can be used to reproduce many of the features of corneal scarring. The expression of other stromal proteins involved in wound healing can now be analyzed in more detail to confirm trends observed in animal models. In addition, soluble and insoluble products can be easily tested as well as innovative therapeutic treatments (drugs, proteins, small interfering RNAs, autologous limbal stem cells, etc.).
